# Supplementary material for: Structural basis of proton-coupled potassium transport in the KUP family
Source: Nat Commun. 2020 Jan 31;11:626. doi: 10.1038/s41467-020-14441-7 (PMC6994465; doi:10.1038/s41467-020-14441-7)
Supplement: Supplementary file 1 — Supplementary Information [file 41467_2020_14441_MOESM1_ESM.pdf]

SUPPLEMENTARY INFORMATION

Structural basis of proton-coupled potassium transport in the KUP family

Igor Tascón[1]*, Joana S. Sousa[2]*, Robin A. Corey[3], Deryck J. Mills[2], David Griwatz[1], Nadine Aumüller[1], Vedrana Mikusevic[1], Phillip J. Stansfeld[3,4], Janet Vonck[2#], Inga Hänelt[1#]

[1]Institute of Biochemistry, Goethe University Frankfurt, Frankfurt am Main, Germany

[2]Department of Structural Biology, Max Planck Institute of Biophysics, Frankfurt am Main, Germany

[3]Department of Biochemistry, University of Oxford, Oxford, UK

[4]School of Life Sciences & Department of Chemistry, University of Warwick, Coventry, CV4 7AL, UK.

*equal contribution, [#]corresponding authors

Supplementary Table 1: Cryo-EM data collection, refinement and validation statistics

	KimA inward-occluded state (EMDB-10092) (PDB 6S3K)
**Data collection and processing**	
Magnification	130,000
Voltage (kV)	300
Electron exposure (e–/Å^2)	70
Defocus range (μm)	-0.5 to -3.2
Pixel size (Å)	1.077
Symmetry imposed	C2
Initial particle images (no.)	2,043,209
Final particle images (no.)	149,724
Map resolution (Å)	3.7
FSC threshold	0.143
Map resolution range (Å)	3 to 6
**Refinement**	
Initial model used (PDB code)	-
Model resolution (Å)	3.95
FSC threshold	0.5
Map sharpening *B* factor (Å^2)	-154
Model composition	
Non-hydrogen atoms	8896
Protein residues	1146
Ligands	6
B factors (Å^2)	
Protein	95.8
Ligand	71.5
R.m.s. deviations	
Bond lengths (Å)	0.39
Bond angles (°)	0.63
Validation	
MolProbity score	1.88
Clashscore	5.76
Poor rotamers (%)	0.62
Ramachandran plot	
Favored (%)	89.33
Allowed (%)	10.67
Disallowed (%)	0

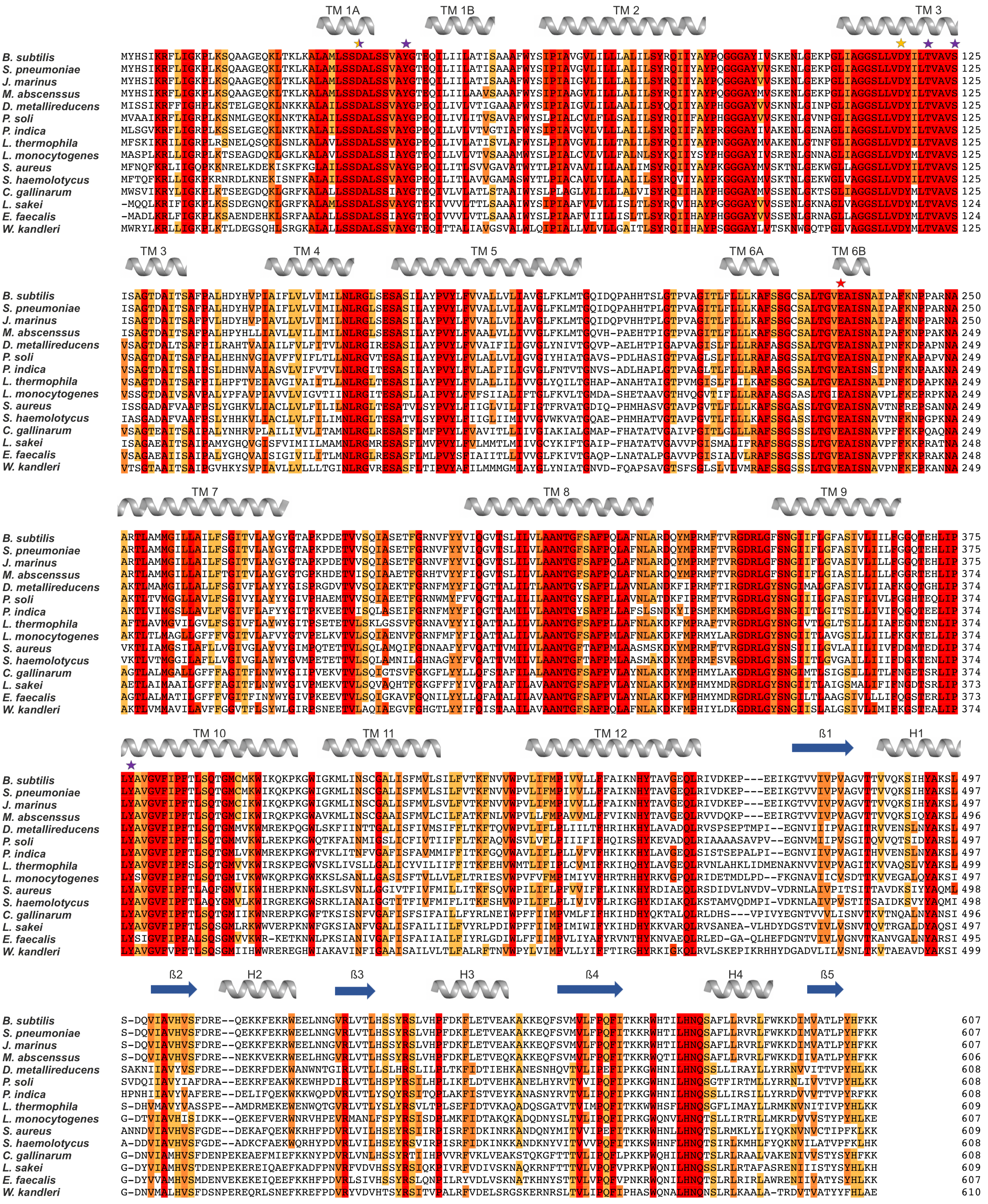

Supplementary Fig. 1: Sequence alignment of KimA from *B. subtilis* with other homologous proteins from different bacteria. Sequences of KimA from *Streptococcus pneumoniae*, *Jeotgalibacillus marinus*, *Mycobacteriodes abscenssus*, *Desulfitobacterium metallireducens*, *Planomicrobium soli*, *Paenisporosarcina indica*, *Lihuaxuella thermophila*, *Listeria monocytogenes*, *Staphylococcus aureus*, *Staphylococcus haemolyticus*, *Carnobacterium gallinarum*, *Lactobacillus sakei*, *Enterococcus faecalis* and *Weissella*

kandleri are aligned. Selection of sequences was done with BLAST and the alignment was performed with T-coffee. Sequence conservation is shown by coloring, red being fully conserved. The secondary structure of KimA is shown above the alignment. The red star indicates a residue potentially implicated in proton coupling, the purple stars indicate residues potentially involved in potassium binding within the substrate binding site, the orange star indicates a residue potentially involved in potassium binding within the intracellular tunnel and the half purple, half orange star indicates a residue involved in potassium binding in both the substrate binding site and within the intracellular tunnel. The figure was prepared using Jalview.

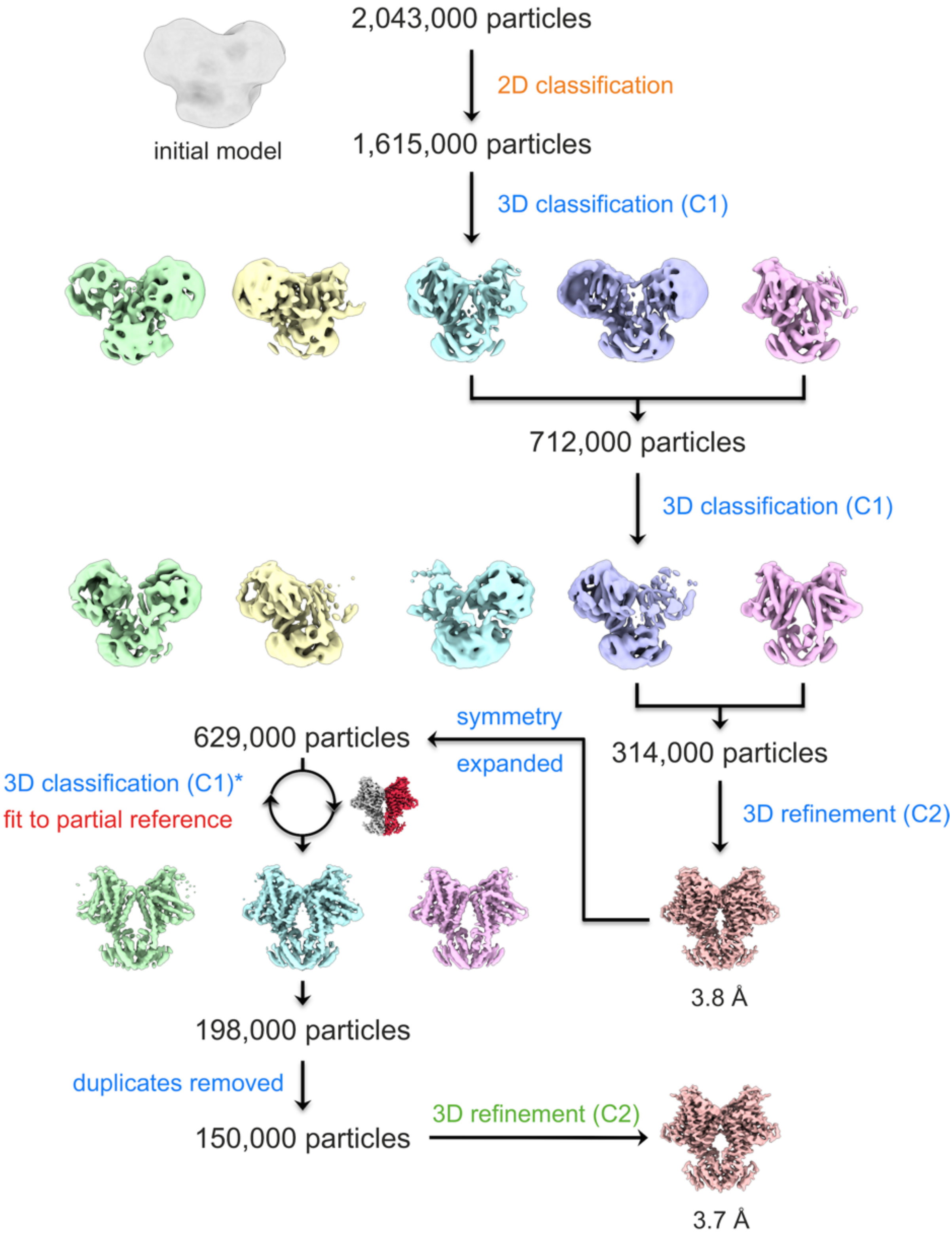

Supplementary Fig. 2: Image processing workflow. Approximately 2,000,000 particles were autopicked using Gautomatch and used for reference-free 2D classification in Sphire. Particles from the best 2D classes were further classified in 3D in Relion, using a low-resolution map generated by the stochastic gradient descent method implemented in Relion

as an initial reference. After two rounds of 3D classification, a set of approximately 300,000 particles was refined in Relion to 3.8 Å resolution, with poor densities at the periphery of the map due to small differences in the relative position of the monomers. In order to obtain a more homogeneous subset of particles, a 3D classification without alignment was performed with a symmetry expanded dataset (reference map shown in grey/red); the position of one membrane domain and the cytoplasmic domain located below was fixed by refitting the maps in Chimera to a partial map (red) every five iterations. This approach made it possible to classify the particles based on the position of the second half of the map, improving the densities at the periphery of the cytoplasmic domain. After reversing the symmetry expansion applied, the best 3D class contained 150,000 unique particles, which produced a map at an overall resolution of 3.7 Å after homogeneous refinement in cryoSPARC. Processes performed in Relion, Sphire, cryoSPARC and Chimera are indicated in blue, orange, green and red, respectively.

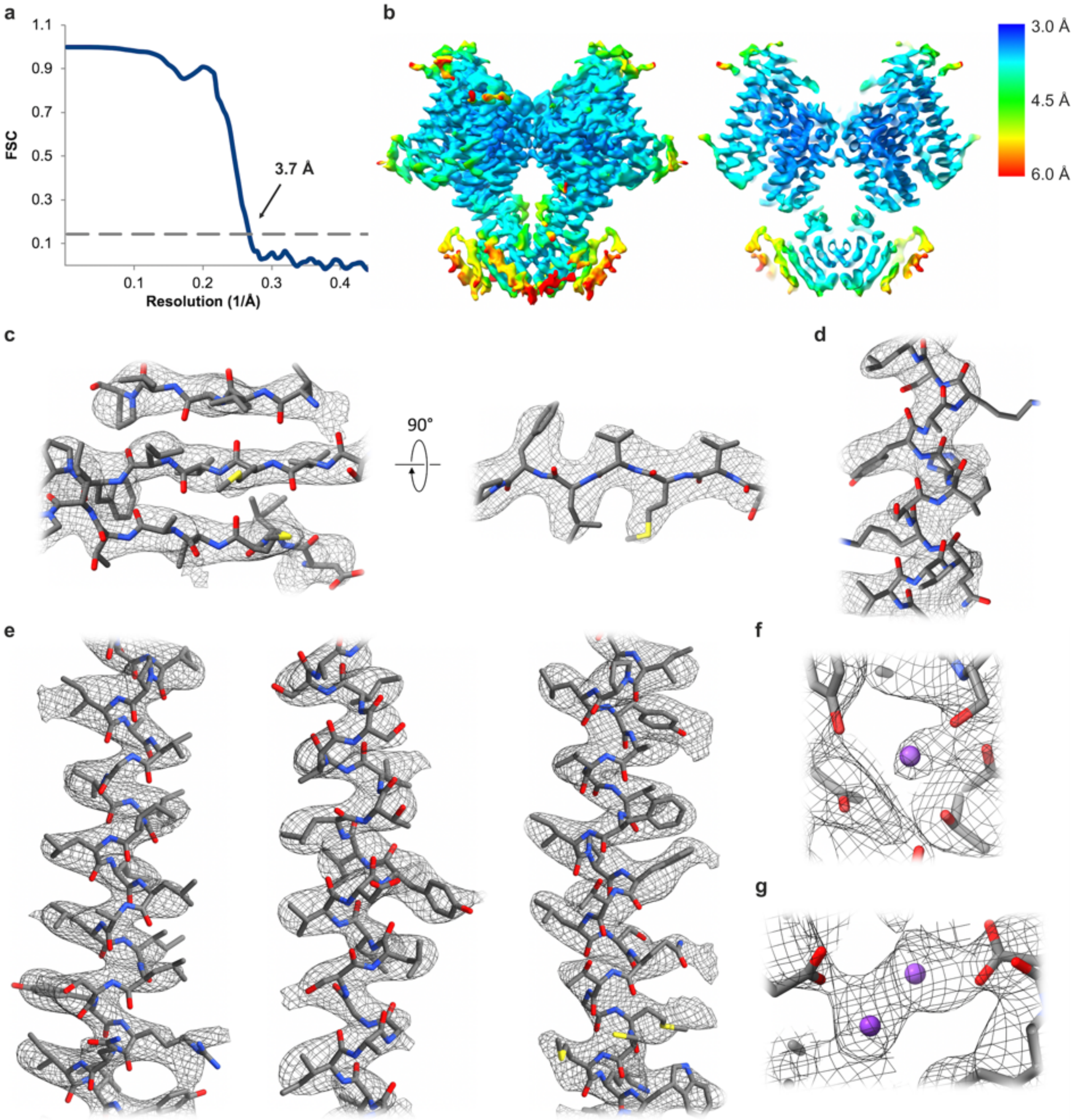

Supplementary Fig. 3: Quality of the cryo-EM map. a, Gold-standard FSC plot for the final refined map. **b**, KimA map colored according to local resolution determined in cryoSPARC, as seen from the membrane (left) and at a central section (right). **c**, Cryo-EM density of a β-sheet with fitted model, showing well-resolved strands and good side chain densities. **d-e**, Cryo-EM density of an α-helix in the cytoplasmic domain (d) and of TM helices (e) with fitted model, showing clear helical pitch and good side chain densities. **f-g**, Cryo-EM densities of the potassium ions in the binding site (f) and the cytoplasmic tunnel (g), in a map low-passed filtered to 3.3 Å.

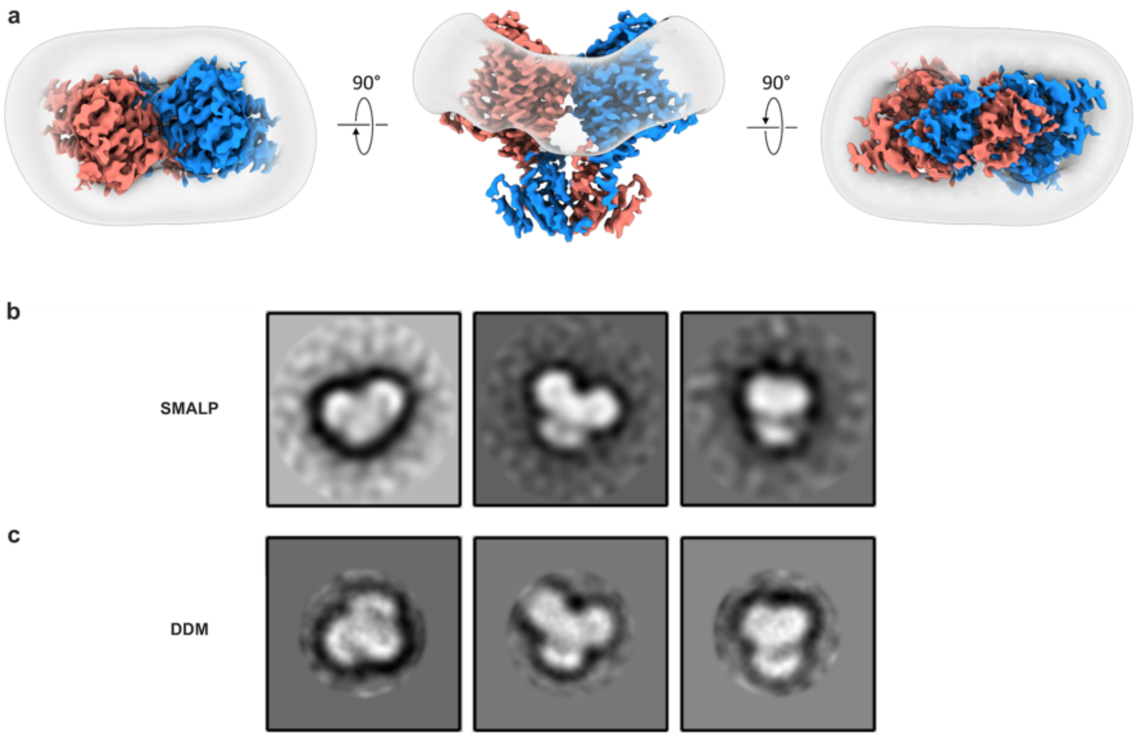

Supplementary Fig. 4: Dimeric arrangement of KimA. **a**, Cryo-EM map of KimA with the SMALP belt, seen from the extracellular side (left), the membrane (middle), and the cytosol (right). The two monomers are depicted in salmon and blue and the SMALP density low-pass filtered to 30 Å is shown in light grey. **b**, **c**, Negative staining 2D class averages of KimA in SMALP and DDM. The overall shape of the dimer is similar when solubilized with SMAs or with detergent and the angle between the membrane domains of both monomers is preserved (middle classes).

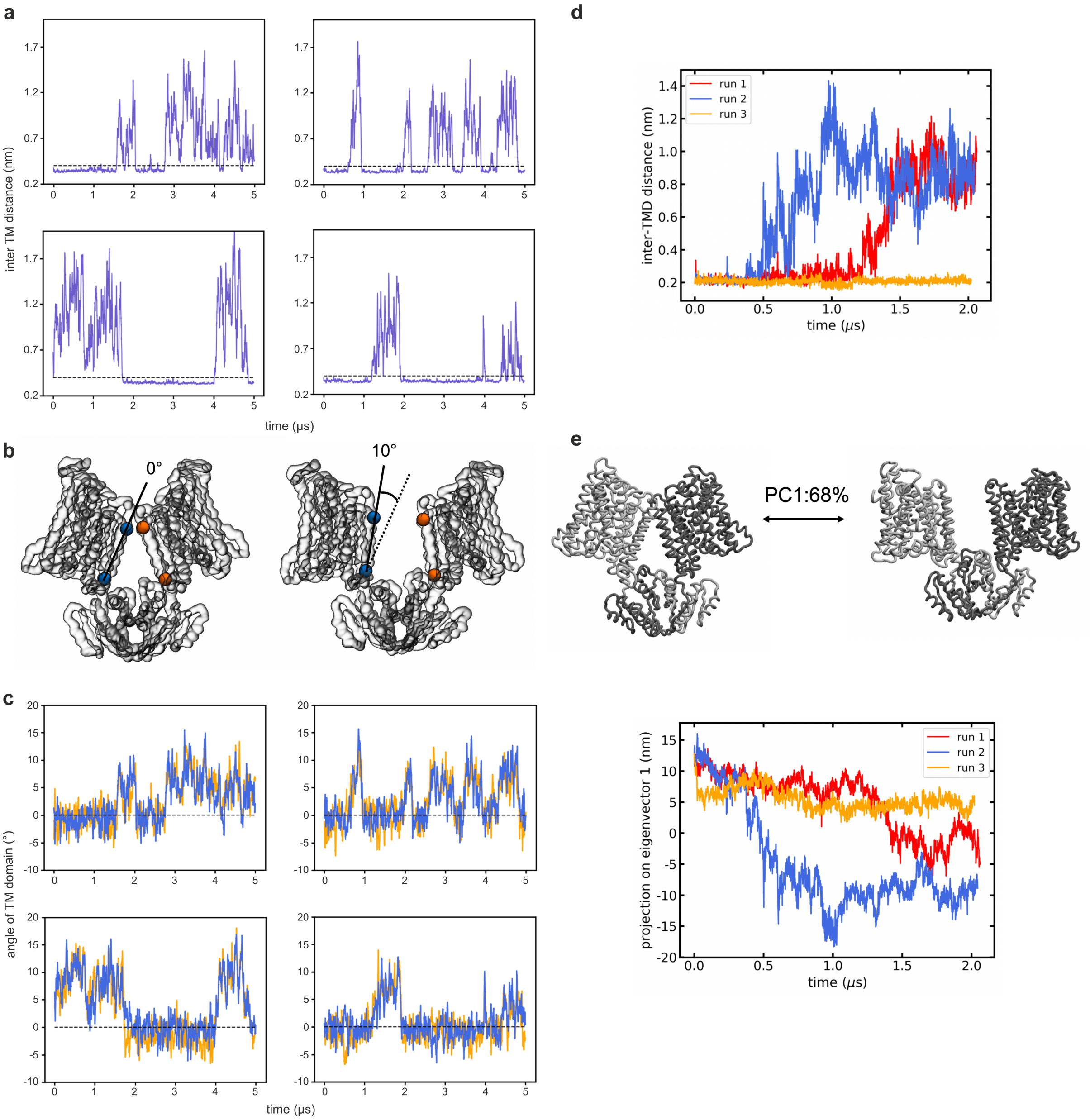

Supplementary Fig. 5: Molecular dynamics simulation of KimA in a lipid bilayer. **a**, Minimum distances between TM domains of each KimA dimer over 4 CG simulations. A dotted line denotes the threshold for contact in the Martini force field. **b**, Calculating the angles of the KimA TM domains for the data in Fig. 3b and panel c of this figure. The protein backbone is shown in white, and the backbone beads of Gly347 and Ile364 of each monomer are shown in blue or orange. The vector between these beads for each monomer was calculated and compared to the cryo-EM structure (set to 0°). **c**, Plots of the TM domain angle over time for the CG simulations, as in Fig. 3b. **d**, As panel a, but minimum distances for 3 atomistic simulations of *ca.* 2 µs. 2 out of 3 runs sample the upright dimer state. **e**, Principal component analysis on the atomistic KimA data. 68% of the total variance can be

described using just PC1 (grey ribbon), corresponding to the opening seen in panel d. Below, each simulation's progression is plotted along PC1.

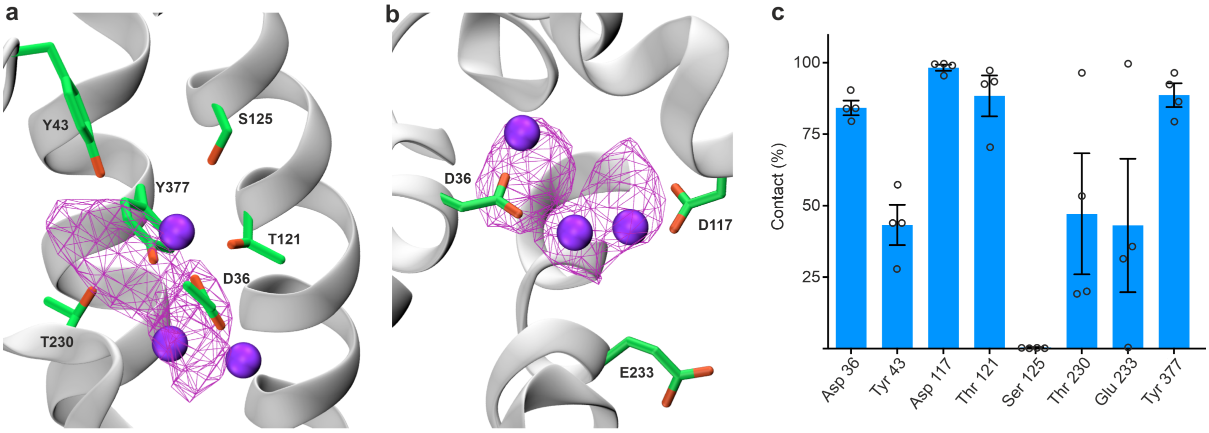

Supplementary Fig. 6: Density of potassium ions over two 135 ns atomistic simulations. The purple mesh shows the K^+ ion densities over the course of the simulations, as computed with VMD's VolMap utility[1]. Purple spheres indicate the potassium ions as found within the EM map. Views are as Fig. 4d-e. **c,** Contact between potassium ions with surrounding residues over the course of two 135 ns atomistic simulations. Contact defined as % of frames where residue-K^+ distance is less than 0.4 nm. n=4 repeats, contacts from individual simulations as dot plots.

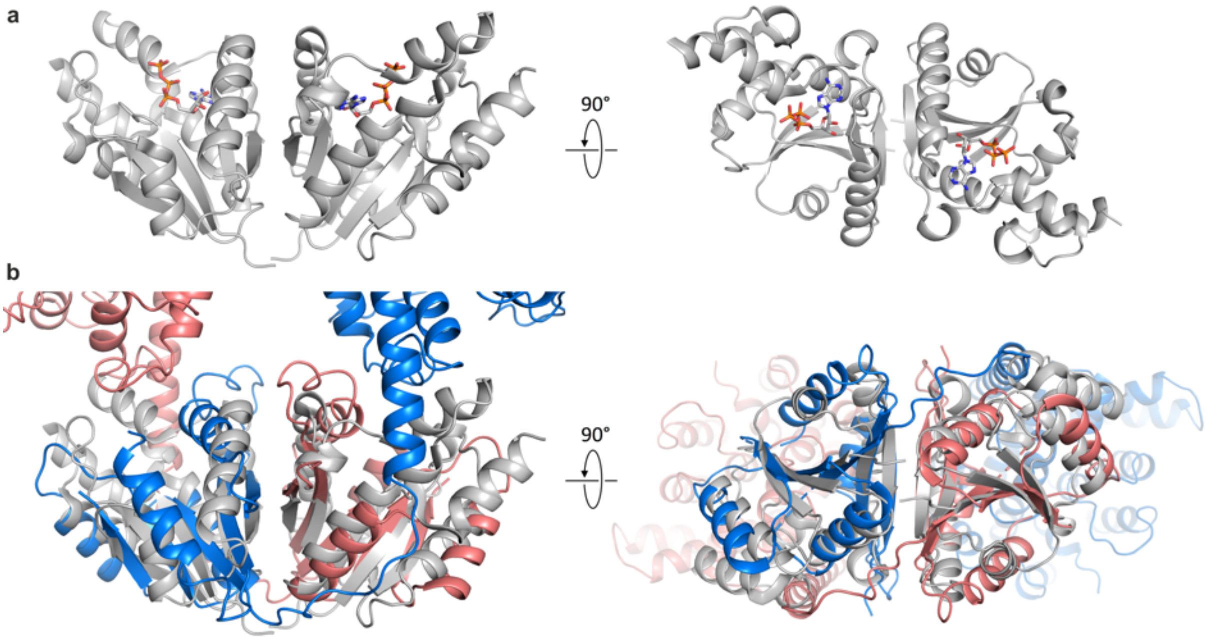

Supplementary Fig. 7: The cytoplasmic domain of KimA resembles the folding of PPAT. **a**, Cartoon representation of the phosphopantetheine adenylyltransferase (PPAT) (grey, 1GN8). Depicted in sticks are the ATP molecules. **b**, Structural comparison of the cytoplasmic domains of KimA (salmon and blue) and the PPAT (grey, 1GN8), with an RMSD of 3.7 Å.

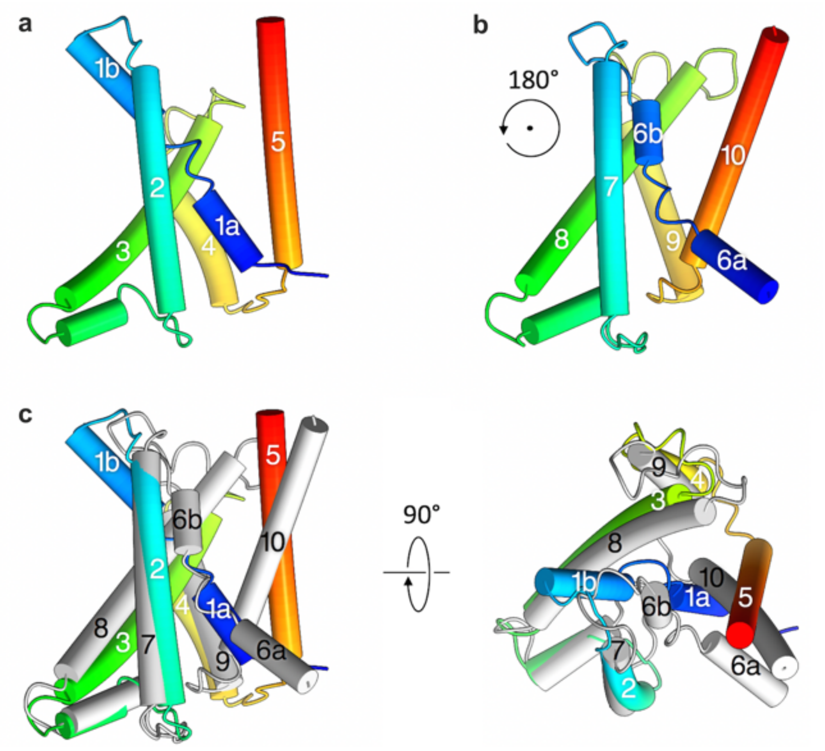

Supplementary Fig. 8: KimA shows a LeuT fold. KimA structural repeats from **a**, TM helices 1 to 5 and **b**, TM 6 to 10 with discontinuities in TM helices 1 and 6, typical of the LeuT fold. TM helices are colored from blue to red. The second repeat was rotated 180° around the symmetry axis. **c**, Side view (left) and top view (right) of repeat 1 (rainbow) superimposed with repeat 2 (grey).

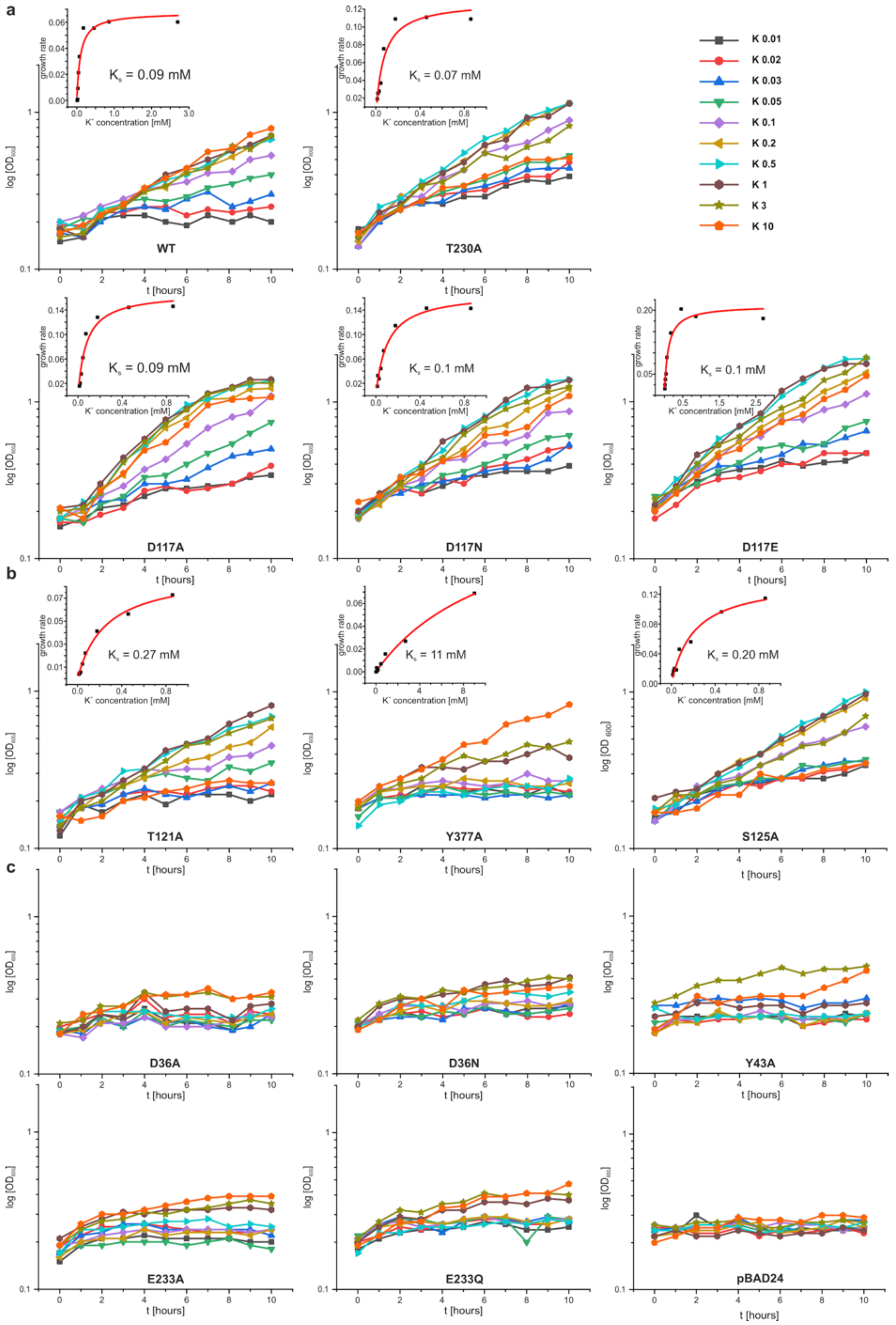

Supplementary Fig. 9: Activity of KimA variants in a heterologous complementation assay. *E. coli* strain LB2003 was transformed with plasmids encoding KimA wild-type or variants and growth at different potassium concentrations, ranging from 0.01 to 10 mM, was

assessed over 10 h. The growth rates μ were determined and plotted against the potassium concentrations. The concentration of the half maximal growth K_S for each KimA variant was determined by fitting with the Monod equation[2]**.** **a**, KimA wild-type and variants that did not affect cell growth. **b**, KimA variants that led to reduced cell growth. **c**, KimA variants that were unable to restore cell growth. Source data are provided as a Source Data file.

a

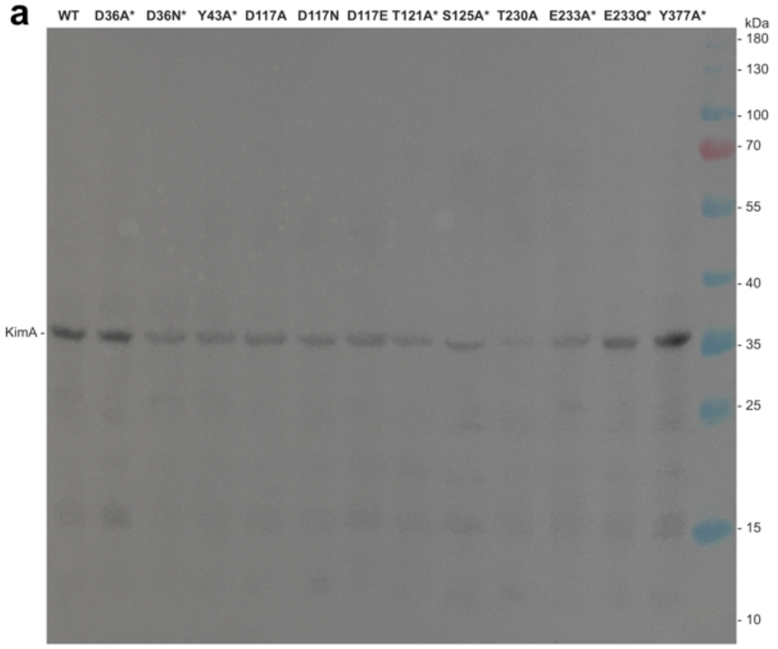

b

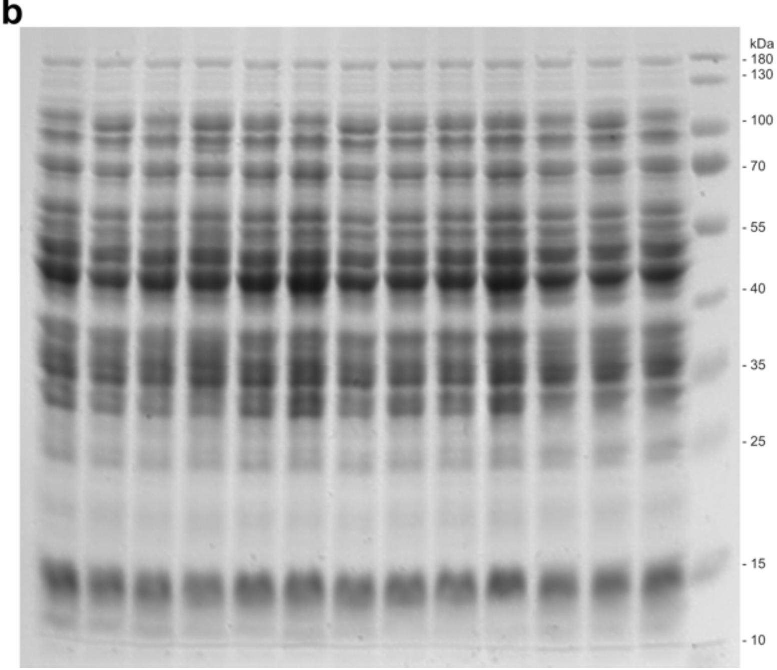

Supplementary Fig. 10: Western blot analysis of the production of KimA wild-type and variants. a, His-tagged KimA variants were detected using an anti-polyHis antibody and a goat anti-mouse IgG HRP-conjugated antibody. All variants were produced in similar amounts, slight variations were not responsible for the lack of activity, since the least expressed $KimA_{T230A}$ was as active as the wild-type. **b**, SDS-PAGE stained with Coomassie and used as a loading control, which is equivalent to the one used for transferring to the blot.

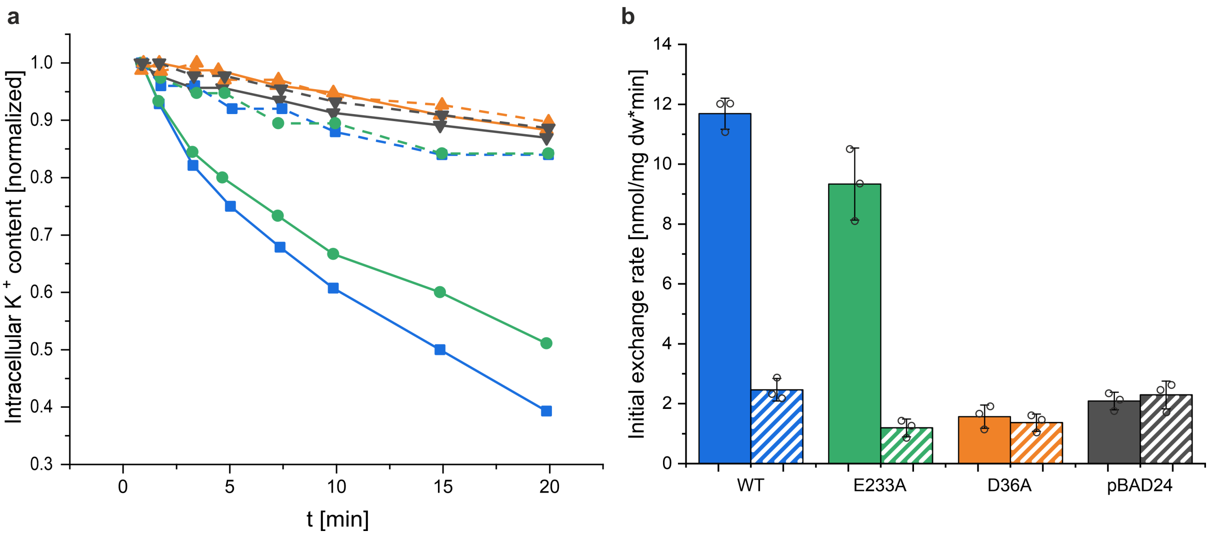

Supplementary Fig. 11: K$^+$/Rb$^+$ exchange via KimA into potassium-loaded *E. coli* LB2003. **a**, Time courses of the K$^+$/Rb$^+$ exchange of cells transformed with plasmids encoding wild type KimA (blue), $KimA_{E233A}$ (green), $KimA_{D36A}$ (orange) or with the empty vector pBAD24 (black). Solid lines represent experiments performed in the presence of 50 mM external Rb$^+$. Dotted lines are negative controls in the presence of 50 mM external Na$^+$. n=3 independent experiments; a representative experiment is shown. **b**, Initial rates of K$^+$/Rb$^+$ exchange given as means of three independent experiments; errors shown are s.d., individual exchange rates as dot plots. Full bars correspond to experiments performed in the presence of 50 mM external Rb$^+$; striped bars represent negative controls in the presence of 50 mM external Na$^+$. Source data are provided as a Source Data file.

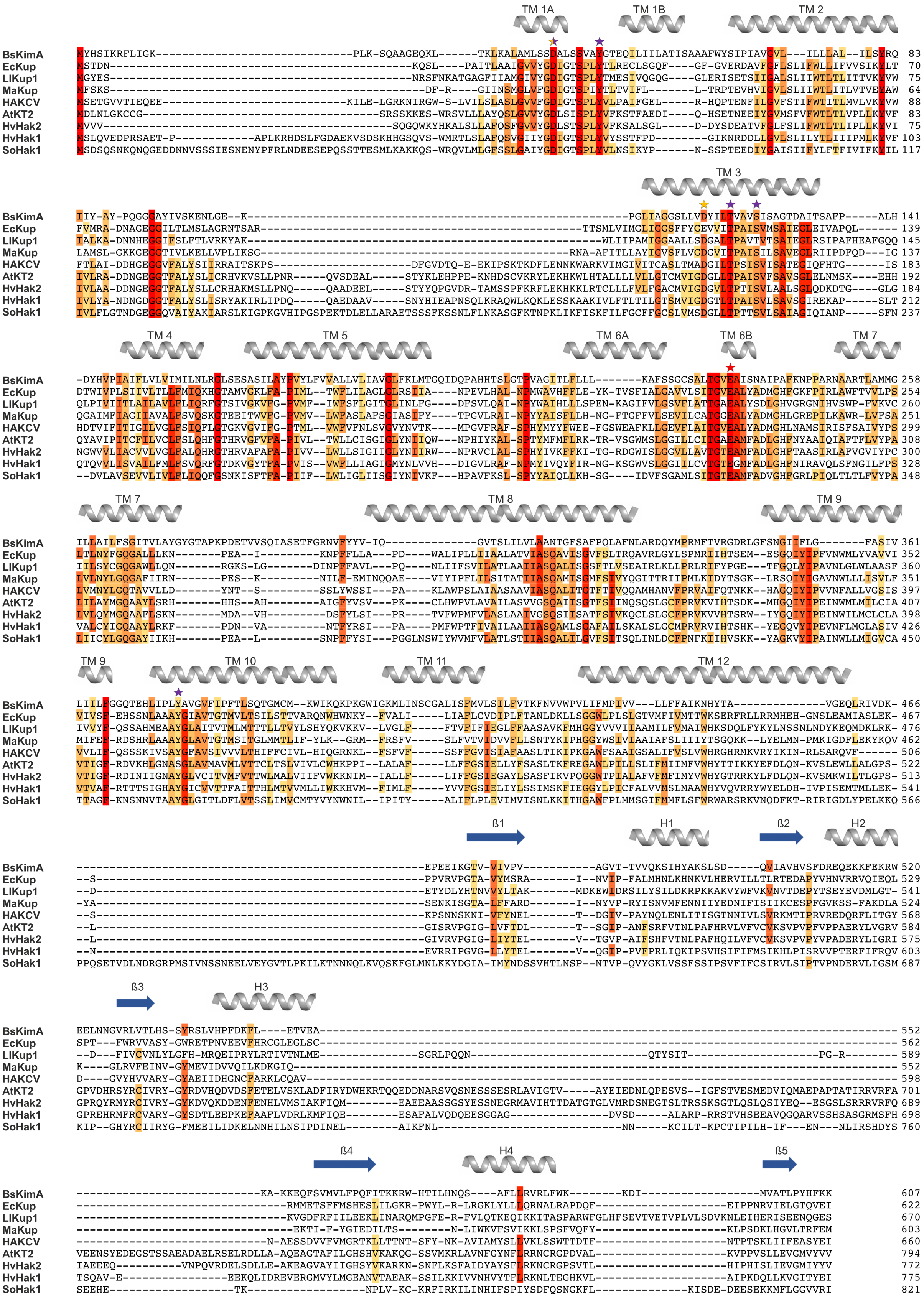

Supplementary Fig. 12: Model-based sequence alignment of KimA with transporters belonging to the KUP family. KUP family members from bacteria (*Escherichia coli,*

Lactococcus lactis), archaea (*Methanosarcina acetivorans*), virus (*Paramecium bursaria, Chlorella virus*), plants (*Arabidopsis thaliana, Hordeum vulgare*) and fungi (*Schwanniomyces occidentalis*) are aligned. The model-based sequence alignment was done using Promals3d server. Sequence conservation is shown by coloring, red being fully conserved. The secondary structure of KimA is shown above the alignment. The red star indicates a residue potentially implicated in proton coupling, the purple stars indicate residues potentially involved in potassium binding within the substrate binding site and the orange star indicates residues potentially involved in potassium binding in the intracellular tunnel, and the half purple, half orange star indicates a residue involved in potassium binding in both the substrate binding site and within the intracellular tunnel. The figure was prepared using Jalview.

Supplementary Methods: List of primer pairs used for cloning in this article.

Cloning of *kimA* into vector pB24C3H:

bsKimApBC3H-RFfw

5'-GGGCTAGCAGGAGGAATTCACCATG TATCATTCAATCAAACGTTTTTTGATTGGG

bsKimApBC3H-RFrv

5'-GTGGACCTTGAAACAAAACTTCTAACTTTTTAAAATGATACGGCAGTGTGGC

Introduction of point mutations:

D36A_For

5'-GCTTTCCTCAGCTGCGCTGTCATCTGTC

D36A_Rev

5'-GACAGATGACAGCGCAGCTGAGGAAAGC

D36N_For

5'-GCTTTCCTCAAATGCGCTGTCATCTGTC

D36N_Rev

5'-GACAGATGACAGCGCATTTGAGGAAAGC

D117A_For

5'-GCGGTTCATTGCTTGTTGCTTATATTTTAACAG

D117A_Rev

5'-CTGTTAAAATATAAGCAACAAGCAATGAACCGC

D117N_For

5'-GCGGGCGGTTCATTGCTTGTTAATTATATTTTAACAG

D117N_Rev

5'-CTGTTAAAATATAATTAACAAGCAATGAACCGCCCGC

D117E_For

5'-GCGGGCGGTTCATTGCTTGTTGAATATATTTTAACAG

D117E_Rev

5'-CTGTTAAAATATATTCAACAAGCAATGAACCGCCCGC

T121A_For

5’-GCTTGTTGATTATATTTTAGCAGTAGCGGTAAGTATTTCC

T121A_Rev

5’-GGAAATACTTACCGCTACTGCTAAAATATAATCAACAAGC

T230A_For

5’-GGATGCTCAGCGTTGGCCGGGGTTG

T230A_Rev

5’-CAACCCCGGCCAACGCTGAGCATCC

E233A_For

5’-TGACCGGGGTTGCGGCCATTTCTAA

E233A_Rev

5’-TTAGAAATGGCCGCAACCCCGGTCA

E233Q_For

5’-GACCGGGGTTCAGGCCATTTCTAATGC

E233Q_Rev

5’-GCATTAGAAATGGCCTGAACCCCGGTC

Y377A_For

5’-CTTAATCCCGTTAGCTGCTGTGGGCGTATTTATTCC

Y377A_Rev

5’-GGAATAAATACGCCCACAGCAGCTAACGGGATTAAG

Y43A_For

5’-GCTGTCATCTGTCGCAGCTGGGACAGAAC

Y43A_Rev

5’-GTTCTGTCCCAGCTGCGACAGATGACAGC

S125A_For

5’-CAGTAGCGGTAGCTATTTCCGCAGGC

S125A_Rev

5’-GCCTGCGGAAATAGCTACCGCTACTG

SUPPLEMENTARY REFERENCES

1 Humphrey, W., Dalke, A. & Schulten, K. VMD: visual molecular dynamics. *J Mol Graph* **14**, 33-38, 27-38 (1996).

2 Monod, J. The growth of bacterial cultures. *Annu. Rev. Microbiol.* **3**, 371-394, doi:10.1146/annurev.mi.03.100149.002103 (1949).
