# Supplementary material for: Structural basis of proton-coupled potassium transport in the KUP family
Source: Nat Commun. 2020 Jan 31;11:626. doi: 10.1038/s41467-020-14441-7 (PMC6994465; doi:10.1038/s41467-020-14441-7)
Supplement: Supplementary file 3 — Reporting Summary [file 41467_2020_14441_MOESM3_ESM.pdf]

nature research

Corresponding author(s): Janet Vonck, Inga Hänelt

Last updated by author(s): Dec 27, 2019

Reporting Summary

Nature Research wishes to improve the reproducibility of the work that we publish. This form provides structure for consistency and transparency in reporting. For further information on Nature Research policies, see Authors & Referees and the Editorial Policy Checklist .

Statistics

For all statistical analyses, confirm that the following items are present in the figure legend, table legend, main text, or Methods section.

n/a	Confirmed	
☐	☒	The exact sample size (*n*) for each experimental group/condition, given as a discrete number and unit of measurement
☐	☒	A statement on whether measurements were taken from distinct samples or whether the same sample was measured repeatedly
☒	☐	The statistical test(s) used AND whether they are one- or two-sided<br>*Only common tests should be described solely by name; describe more complex techniques in the Methods section.*
☒	☐	A description of all covariates tested
☒	☐	A description of any assumptions or corrections, such as tests of normality and adjustment for multiple comparisons
☐	☒	A full description of the statistical parameters including central tendency (e.g. means) or other basic estimates (e.g. regression coefficient) AND variation (e.g. standard deviation) or associated estimates of uncertainty (e.g. confidence intervals)
☒	☐	For null hypothesis testing, the test statistic (e.g. *F*, *t*, *r*) with confidence intervals, effect sizes, degrees of freedom and *P* value noted<br>*Give P values as exact values whenever suitable.*
☒	☐	For Bayesian analysis, information on the choice of priors and Markov chain Monte Carlo settings
☒	☐	For hierarchical and complex designs, identification of the appropriate level for tests and full reporting of outcomes
☒	☐	Estimates of effect sizes (e.g. Cohen's *d*, Pearson's *r*), indicating how they were calculated

Our web collection on statistics for biologists contains articles on many of the points above.

Software and code

Policy information about availability of computer code

Data collection	EPU (Thermo Scientific), CG Martini force field using the 321 open beta 3.0.b.3.2, MemProtMD protocol, Gromacs 2019
Data analysis	T-coffee, Promals3d server, Jalview, EMAN boxer, Relion 2.1, MotionCor2, CTFFIND4, ISAC (within Sphire), Gautomatch (by Kai Zhang, MRC---LMB), USCF Chimera 1.13, Pymol 2.11, Relion 3.0, Phyre2 server, Coot 0.8.2, Phenix.real_space_refinement, MolProbity, Hollow 1.2, HOLE 2, MCCE program, VMD, Gromacs 2019

For manuscripts utilizing custom algorithms or software that are central to the research but not yet described in published literature, software must be made available to editors/reviewers. We strongly encourage code deposition in a community repository (e.g. GitHub). See the Nature Research guidelines for submitting code & software for further information.

Data

Policy information about availability of data

All manuscripts must include a data availability statement. This statement should provide the following information, where applicable:

- Accession codes, unique identifiers, or web links for publicly available datasets
- A list of figures that have associated raw data
- A description of any restrictions on data availability

The cryo-EM map and model were deposited in the wwPDB with accession codes EMD-10092 and 6S3K, respectively. Other data generated and analysed during this study are included in this published article and its Supplementary Information file. The source data underlying Figs. 1 and 5 and Supplementary Figs. 9 and 11 are provided as a Source Data file. Further data that support the findings of this study are available from the corresponding author upon reasonable request.

Field-specific reporting

Please select the one below that is the best fit for your research. If you are not sure, read the appropriate sections before making your selection.

[x] Life sciences   [ ] Behavioural & social sciences   [ ] Ecological, evolutionary & environmental sciences

For a reference copy of the document with all sections, see nature.com/documents/nr-reporting-summary-flat.pdf

Life sciences study design

All studies must disclose on these points even when the disclosure is negative.

Sample size	A sample size determination does not apply to the described research.
Data exclusions	Obvious outliers and data with poor signal to noise ratio were excluded.
Replication	Functional assays were repeated in at least three independent experiments. Four independent MD simulations each were performed. For all other experiments replications do not apply.
Randomization	n/a
Blinding	n/a

Reporting for specific materials, systems and methods

We require information from authors about some types of materials, experimental systems and methods used in many studies. Here, indicate whether each material, system or method listed is relevant to your study. If you are not sure if a list item applies to your research, read the appropriate section before selecting a response.

Materials & experimental systems

n/a	Involved in the study	
[ ]	[x]	Antibodies
[x]	[ ]	Eukaryotic cell lines
[x]	[ ]	Palaeontology
[ ]	[x]	Animals and other organisms
[x]	[ ]	Human research participants
[x]	[ ]	Clinical data

Methods

n/a	Involved in the study	
[x]	[ ]	ChIP-seq
[x]	[ ]	Flow cytometry
[x]	[ ]	MRI-based neuroimaging

Antibodies

Antibodies used	anti-His antibody from mouse (dilution 1:3000, Sigma-Aldrich, cat.no. H1029), secondary anti-mouse IgG-peroxidase antibody produced in goat (dilution 1:20000, Sigma-Aldrich, cat.no. A2554)
Validation	Monoclonal anti-polyHis antibody from mouse; antibody suitable for immunoblotting, dot blot assays, ELISA and immunocytochemistry. he Monoclonal Anti-polyHistidine (mouse IgG2a) antibody recognizes native or denatured, reduced forms of synthetic polyhistidine and polyhistidine-tagged fusion proteins. The antibody preferentially recognizes the N-terminal tagged fusion protein and is reactive with fusion proteins expressed by the prokaryotic expression vectors pET, pRSET, and pTrc.

Animals and other organisms

Policy information about studies involving animals; ARRIVE guidelines recommended for reporting animal research

Laboratory animals	n.a.
Wild animals	n.a.
Field-collected samples	n.a.
Ethics oversight	n.a.

Note that full information on the approval of the study protocol must also be provided in the manuscript.
